# Your place or mine? Exploring birdwatching tourists’ behaviour disturbing birds in a nature reserve

**DOI:** 10.1007/s10344-023-01678-y

**Published:** 2023-04-11

**Authors:** Øystein Aas, Frida Marie Omma Jørgensen, Stian Stensland, Tone Reiertsen, Hilde Nikoline Hambro Dybsand

**Affiliations:** 1 Faculty of Environmental Sciences and Nature Management, Norwegian University of Life Sciences, 1432 Ås, Norway; 2 Norwegian Institute for Nature Research, 9296 Tromsø, Norway; 3 Department of Sport Sciences, UiT Norwegian Arctic University, Alta, Norway

**Keywords:** Avitourism, Wildlife disturbance, Seabirds, Theory of planned behaviour

## Abstract

To manage for more sustainable wildlife viewing tourism, a better understanding of tourists’ behaviour that might disturb and negatively affect wildlife such as birds is needed. We conducted a qualitative case study of visitors to Hornøya, a protected bird cliff in Northern Norway. Behaviours with the potential to disturb seabirds at the site were explored using the theory of planned behaviour as a guiding framework. In-depth interviews and observations were used to explore why some visitors perform illegal or unwanted, potentially harmful behaviours, and to understand attitudes, social norms, and perceived behaviour control as influencing factors. The tourists visited Hornøya to experience seabirds in their natural surroundings, without causing harm. They were emotionally affected by the bird encounters, which may lead to thoughts and reflections about environmental challenges and nature protection. Visitors generally did not intend to disturb birds during these encounters. However, many visitors interpreted the birds’ behaviour as meaning that they were not easily disturbed. Poorly developed social norms among visitors, in combination with limited presence of surveillance/guide personnel, and strong behavioural control among some visitors (willingness and ability to engage in illegal behaviour) led to occasional breaching of formal rules as well as incidents of inappropriate, potentially disturbing behaviour towards birds. Experienced wildlife photographers tended to show willingness and ability to engage in illegal behaviour more often than the two other segments.

**Supplementary Information:**

The online version contains supplementary material available at 10.1007/s10344-023-01678-y.

## Introduction

Birdwatching tourism is a wildlife-based, fast-growing niche in nature-based tourism (Kronenberg 2016; De Salvo et al. 2020). It may have both positive and negative conservation impacts. Birdwatching tourism can raise awareness of, and be an incentive for bird protection, increase the sustainability of birdwatchers’ behaviour both at the tourism site and in their everyday lives, and generate funding for conservation (Borges de Lima and Green 2017; Şekercioḡlu 2002). Wildlife-based tourism and recreation can, however, also disturb wildlife, causing harm at the individual, population and species level (Green and Giese 2004; Lorentsen and Follestad 2014; Steven and Castley 2013). Birds habituated to human disturbance may become bolder, increasing their vulnerability to predation (Geffroy et al. 2015). Thus, human disturbance can have multiple negative consequences for bird communities and populations (Valentine and Birtles 2004), cause long-term negative effects on the ecosystem (Courchamp et al. 2006), and hinder needed biodiversity conservation. In some cases, birdwatching tourism can pose fundamental threats to critically endangered bird species (Steven et al. 2014).

Despite the potential negative impacts, experts believe that birdwatching tourism can be sustainable and eco-friendly where the positive impacts are larger than the negative ones. In particular, it is suggested that specialised, so-called serious birdwatchers can contribute to conservation through high economic revenue (Şekercioḡlu 2002). However, to achieve birdwatching tourism where negative impacts are reduced as much as possible, Green and Giese (2004) and Fatima and Khan (2017) highlighted the importance of researching tourist interactions with wildlife in order to more efficiently mitigate potential impacts on wild animals and their environments. Similarly, Fennell and Yazdan Panah (2020) underlined that as animal-focused tourism continues to grow, careful attention must be paid to how tourists behave in the natural world. Newsome (2017) emphasized that an increased understanding of how to access and view nature with less disturbance can help foster and maintain sustainability. Manfredo (2008) claimed that better knowledge of social groups dynamics, attitudes, and norms are important to reduce human disturbance of wildlife.

### Segmentation of birdwatching tourists

Wildlife tourists, including birdwatching tourists, are diverse and vary in their behaviour, experience preferences and past experiences (Slater et al. 2019; Dybsand et al. 2021; Stensland et al. 2021). Diversity might also reflect differences in experience and knowledge that might explain why some conduct unwanted behaviours. Birdwatching tourists have been categorised and segmented using different concepts and variables. The most used framework is the three-dimensional concept of specialisation, which considers behaviour, skills and knowledge, and commitment (McFarlane 1994), often distributed along a continuum from beginners and generalists to specialists. Committed and experienced birders have in former studies been separated into birders and photographers (Slater et al. 2019). However, among wildlife photographers, the motives and skills vary from competing in photo contests and selling photos commercially to simple personal enjoyment and sharing photos with family and friends (Cole and Scott 1999; Scott and Shafer 2001).

In this paper, we categorise the visitors into three groups: *generalist birdwatching tourists*, *experienced birdwatchers*, and *experienced bird photographers*. The generic terms ‘birdwatching tourists’ or ‘birdwatchers’ are used when discussing visitors to the study area Hornøya in general.

### Behaviours potentially disturbing seabirds

Tourists can disturb wildlife and act inappropriately (Manfredo 1992; Steven et al. 2014). Ziegler et al. (2018) claims that harmful behaviours of wildlife-watching tourists can result from straightforward ruthlessness or from the ‘guilty pleasure’ of wanting close interactions with wildlife. Such behaviours can also stem from lack of knowledge (Moore et al. 2015; Widner-Ward and Roggenbuck 2003). Illegal behaviour violates society’s formal rules, as codified in laws and regulations, in this case to protect birds, and can result in penalties ranging from fines to more serious punishments such as criminal conviction. Other behaviours, while legal, can still be judged and considered harmful, inappropriate, or undesirable, e.g. from the perspective of an experienced ornithologist, a researcher, a nature-reserve manager or another visitor. Such behaviours that can be harmful might also violate social norms, or be considered undesirable in specific situations or places (Manfredo 1992).

### Theory of planned behaviour

According to the theory of planned behaviour (TPB), human behaviours can be investigated by identifying attitudes towards behaviours and behavioural beliefs, subjective norms and normative beliefs, and perceived behavioural control and control beliefs (Ajzen 1991). Behavioural beliefs can be divided into *instrumental beliefs* and *affective/experiential beliefs*. Instrumental beliefs give insight into an individual’s assessment of perceived positive and negative outcomes from a behaviour (Ajzen 1991). Affective/experiential beliefs are the positive or negative feelings that are associated with the behaviour, and together they form the individual’s *attitudes* towards a behaviour. *Social norms* consist of *injunctive norms*, which concern approval or disapproval of certain behaviours from others, and *descriptive norms*, which are beliefs about what is right and wrong behaviours derived from observing others (Ajzen 1991). Lastly, *control beliefs* involve the influencing factors that discourage or foster a certain behaviour, especially the individual’s *perceived control of performing the behaviour* (Ajzen 2002).

*Attitudes* towards wild animals may also be influenced by anthropomorphism (Manfredo et al. 2020; Curtin 2005), which entails personifying animals and comparing human and animal behaviour. Human attributes are imposed on the observed animals, like a mirror of ourselves.

The term *norm* covers a variety of entities which provide guidelines on how people ought, should, or may behave (Koller 2014). Formal norms are structural regulations set and enforced by authorities where violation can lead to fines or other forms of societal punishment (Heywood 2011). Social norms indicate which behaviours are considered right or wrong in a social group, while personal norms can be understood as self-expectations or feelings of moral obligation activated by factors such as awareness of consequences and situational responsibility (Harland et al. 2007; Heywood 2011). However, a personal norm also often involves the individual’s perception of the social pressure to engage or not engage in a certain behaviour (Ajzen 2001). A person will, in general, perform a behaviour if he/she thinks that the people closest to them would encourage it. Birdwatchers often belong to specific social subgroups with a set of values that promote certain appropriate behaviours around birds, often tending to self-categorise based on a ‘prototype’ group member (Manfredo 2008). Deviation from group norms can lead to social sanctions (reward or punishment), such as verbal communication or body language. If a person’s behaviour contradicts a norm, they can experience negative feelings like guilt and shame. Consequently, norms shape behaviour (Heywood 2002).

While wildlife tourism seems to be growing, and, as far as these authors know, previous research on the potential negative impacts of wildlife tourism in Northern Europe specifically, is limited, and more research is needed that can increase the understanding of why some tourists behave in ways that can disturb and harm wildlife. Therefore, our overall objective for this study is: How can wildlife tourists’ behaviour that can disturb and harm seabirds be understood, using birdwatching at Hornøya, Northern Norway as a case? Using Ajzen’s (1991) theory of planned behaviour as an analytical framework, we explored the following specific research objectives:

1. Identify and characterise occurring illegal and legal but potentially harmful behaviours conducted by birdwatching tourists visiting the Hornøya island, Northern Norway.
2. Explore birdwatching tourists’ attitudes towards disturbing birds, by investigating how they interpret bird behaviour during their visit and how they assess the consequences of potential disturbance.
3. Explore the role of informal norms and perceived behaviour control in regulating the birdwatchers’ behaviour that might disturb birds at Hornøya.

## Materials and methods

### Study area

**Fig. 1:**
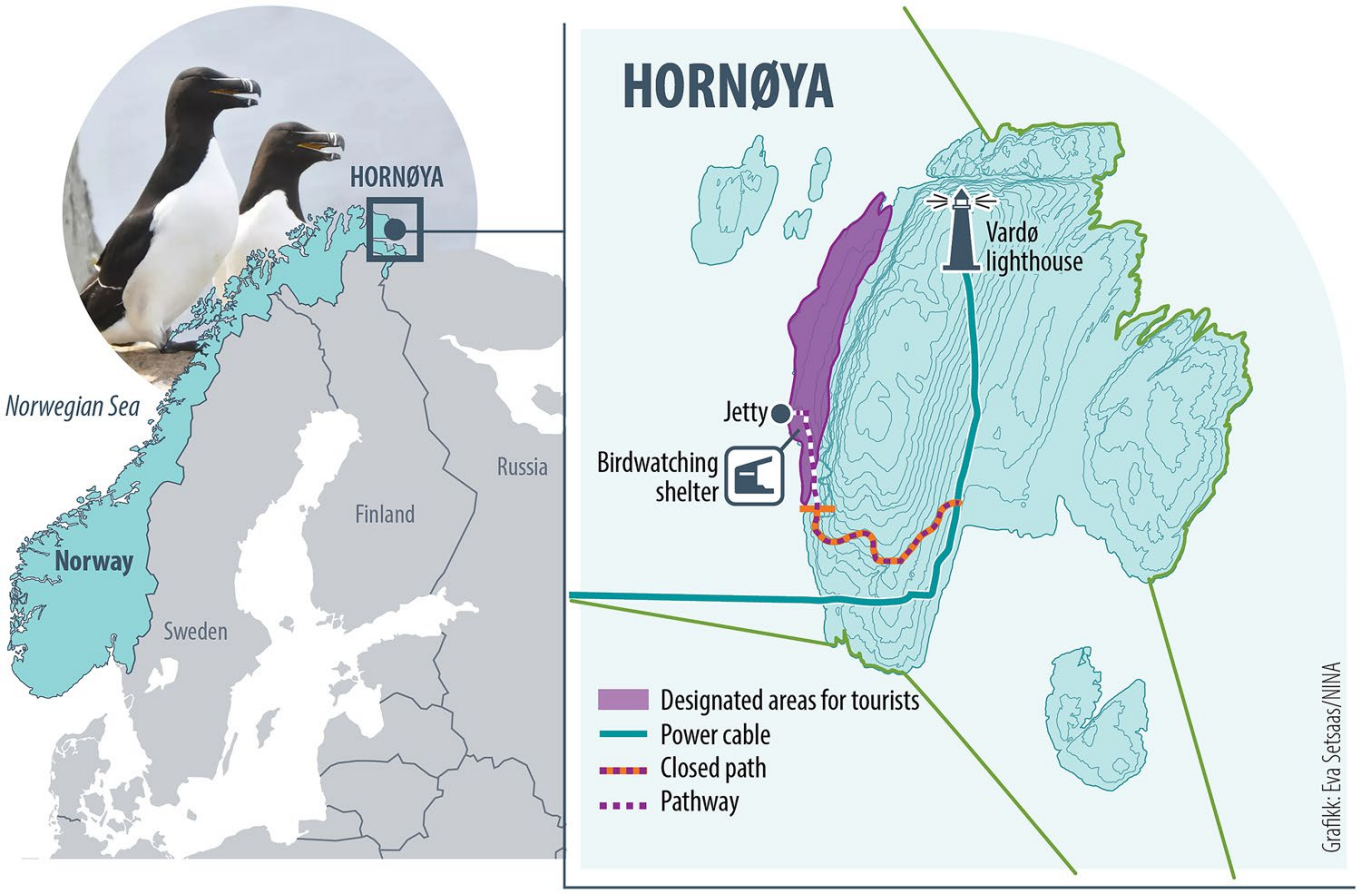
Location of study area at Hornøya, Norway, and detailed map of the bird cliff island, including the border between the visitation area and the prohibited area, the shelter and jetty

Hornøya is an island located by the coast of the Varanger Peninsula in north-eastern Norway (70° 22′ N, 31° 01′ E), just outside the town of Vardø (Fig. 1). Hornøya, as well as the Varanger region, is subject to growing interest from European birdwatching tourists, and, to a lesser extent, birdwatchers from outside of Europe. The Varanger Peninsula is currently considered one of the most successful birdwatching destinations in Norway, and the Hornøya bird cliff is often described as the most spectacular site in Varanger. The island is occupied by more than 80,000 colony-breeding birds, and several Arctic seabird species on the International Union for Conservation of Nature’s Red List with declining populations nest there (Henriksen and Hilmo 2015), including the common guillemot (*Uria aalge*), Brünnich’s guillemot (*Uria lomvia*), black-legged kittiwake (*Rissa tridactyla*), and Atlantic puffin (*Fratercula arctica*). Research-based observations suggest that when located close to the tourist areas on Hornøya, some of these vulnerable species are disturbed by birders (Reiertsen et al. 2018). Additionally, since 2018, breeding success has been low for birds nesting on open ledges, due in part to anti-predator behaviour in response to white-tailed eagle (*Haliaeetus albicilla*) presence at the bird-cliff, and egg-predation by larger gulls and ravens (Reiertsen pers. comm.).

**Fig. 2:**
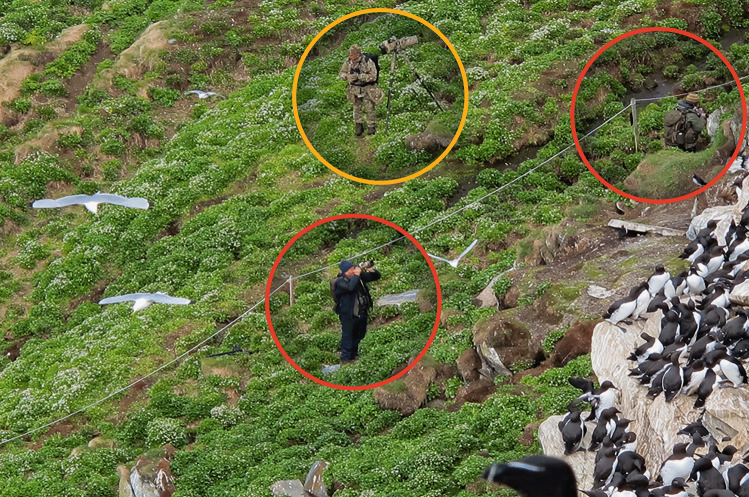
Observation showing two visitors (marked with red circles) violating the rules by crossing the boundary rope demarcating the visitation area from the protected area, while another individual watches from outside the boundary (marked with yellow). Photo: Frida M. O. Jørgensen

The island is protected as a nature reserve, with tourist visitation limited to small parts of the island during the breeding season from March 1 to August 15 (see Fig. 1 for details). In the visitation area, there is a shelter. Due to the small area open to visitors, they mostly stay near the shelter. Birds also are present in the visitation area, nesting there or commuting through the area on their way from the nests to the sea and back. The island is only accessible by boat, and nearly all visitors access it via organised boat transportation from Vardø harbour (round trip fare in 2018 was 400 NOK/approximately 40 euros; in 2021, it was 600 NOK). The trip takes around 15 min one way. Some tour operators also offer guiding at the site, and a few tourists attend more exclusive trips with small boats and activities such as snorkelling among the seabirds, but a majority of visitors are on self-organised trips, dependent on the organised shuttle boat from Vardø. Information about the bird cliff and the reserve’s regulations, such as where tourists are allowed to visit, is limited to basic signs and simple rope fences (see also Fig. 2).

Registered birdwatching tourist visitations to Hornøya have nearly doubled over the last several years, from 1100 visitors in 2012 to 1930 in 2019 (the COVID-19 pandemic reduced visitation in 2020 and 2021, and figures for 2022 are not yet available). As is often the case for colonial cliff-breeding bird species, birds at Hornøya allow humans to approach closely, likely due to several mechanisms including anti-predator strategies and costs associated with leaving the nest (Ellenberg 2017). The opportunity for close encounters with birds combined with the presence of rare Arctic species, large numbers, and species diversity are key reasons why Hornøya is a highly attractive site to birdwatchers (Dybsand et al. 2021).

### Data collection

We collected data on birdwatching tourists’ behaviour at Hornøya via semi-structured, qualitative interviews and systematic observations on site. Fieldwork was conducted over 20 days during the prime birdwatching season, from mid-May to the end of June 2018.

We applied a simple categorisation of the visitors partly inspired from specialisation theory to classify the birdwatching tourists at Hornøya into *three groups* in line with the approach of Slater et al. (2019) as either:

- *generalist birdwatching tourist*
- *experienced birdwatcher*
- *experienced bird photographer*

Visitors were classified into one of these categories by the observer/interviewer at the site, based on a simple assessment of their clothing and equipment (e.g. the use of and type of binoculars and telescopes, cheap or expensive/specialised cameras, use of tripods, specialist clothing) and their behaviour (vocal and bodily expressions, taking notes or not, level of patience and concentration), in line with existing research on typical characteristics of experienced and less experienced birdwatching tourists (e.g. Curtin 2010).

#### Semi-structured interviews

We invited birdwatching tourists close to the shelter to participate in interviews, aiming to interview a variety of visitors (different categories of birdwatching tourists and diverse in sociodemographic characteristics).

Overall, we interviewed 61 individuals during 48 interviews. While we aimed to interview individuals alone, we interviewed pairs and groups together if they insisted. Thirty-eight interviews were with individuals, seven were with pairs/couples and three were with groups of three or four. Most interviews were conducted in English. Informants from Scandinavia were interviewed in Norwegian. Interview duration varied between 9 and 39 min, with most interviews lasting 10 to 19 min.

The interview guide covered five main themes (see supplementary material for a complete guide): background information, human-bird interactions, behavioural beliefs (attitudes), normative beliefs (norms), and control beliefs (perceived behavioural control). To explore human-bird interactions, we posed questions such as, ‘Did you expect to have close encounters with the birds?’. To examine behavioural beliefs, questions focused on awareness of the nature reserve’s rules, attitudes towards disturbing birds, and the perceived consequences of disturbance, e.g. ‘What kind of human behaviour do you think can disturb the birds at Hornøya?’. Questions regarding normative beliefs were directed towards the informant’s perception of others approving or disapproving certain behaviours, and potential consequences of not complying with social codes.

#### Observations

In addition to interviews, we conducted direct observations at Hornøya. These observations cannot strictly confirm or reject data collected in the interviews but are an important supplement, particularly regarding social desirability bias (Stewart et al. 2009). We conducted both distant, hidden observations and participatory observations (Veal 2011). Distant, systematic observations were conducted from three locations approximately 15, 40 and 60 m away from the main visitation areas. Observations were sometimes aided by binoculars, and the observer was assumed hidden from visitors. Total distant observation time was approximately 8 h over 5 days (1 to 1.5 h per day, depending on the frequency and timing of arriving boats). Participatory observations were conducted over 16 days and took place in between the interviews. During these periods, the observer stayed present and took on an *incognito* role (Veal 2011), pretending to solely enjoy the views. Observations took place across the whole day and with different numbers of other visitors present.

### Analysis

Illegal and unwanted, potentially harmful behaviours were identified and categorised according to the following system established by the research group:

Illegal behaviours are activities clearly breaching the rules for the bird reserve:

- Crossing ropes that mark the border between the designated visitor area and the strict reserve where humans are not allowed during the breeding season (see example in Fig. 2).
- Walking past signs that delimit the area between tourist visitors and birds.
- Walking past signs as well as crossing border ropes during one incident.

Behaviours that the research team assessed as having a potentially harmful disturbance effect on the sea birds:

- Loud noises and abrupt movements near birds in the visitation area.
- Staying close to a colony if birds were resting within the visitation area.
- Moving a camera lens close to a bird that was inside the visitation area.
- General disturbance of birds nesting near the birdwatching shelter.
- Leaving a trace by dropping or leaving behind litter/waste.

Verbatim transcriptions of the sound-recordings from interviews and field memos were analysed in ATLAS.ti 8 (Scientific Software Development 2002–2019). Use of this qualitative data analysis tool facilitated coding, categorization and identifying key meanings and discourses. Additionally, networks of code groups effectively identified linkages between the empirical results (Sirakaya-Turk et al. 2017). Emergent patterns enabled identification of the most relevant discourses and associated quotations from the interviews, in line with theory and to reject or support theoretical assumptions in a hermeneutical manner (Kvale and Brinkmann 2015).

### Ethical considerations

Since no person-identifying data were collected, the study did not need formal approval from the Norwegian Centre for Research Data.^1^ All the interviewed informants were informed of the study’s aim and gave consent. We ensured that all birdwatchers were unrecognizable in all photographs. Distant observations conducted without participants’ knowledge and consent, such as those carried out in this study, do raise ethical concerns regarding those individuals’ rights to privacy. According to approved Norwegian guidelines for ethical social research, this is acceptable if there is no direct contact between the researcher and those being researched, given that the data is not considered sensitive and cannot lead to identification and that the research is clearly useful for society (NESH 2021). Overall, we judge that the information gained from these anonymous, hidden observations justify this approach. The researchers never acted as authority figures, e.g. rangers or managers, and never corrected or otherwise reacted to illegal or potentially harmful behaviour.

## Results and analysis

### Overview of informants

Ten of the 61 informants interviewed for this study had visited Hornøya previously. Approximately three of four (47 of 61) informants were men (Table 1). Age was not formally recorded, but most of the informants were judged to be between 40 and 65 years of age. Table 1 also presents informants’ classification as either ‘generalist birdwatcher’, ‘experienced birdwatcher’ or ‘experienced bird photographer’ by their country/region of origin. Most informants were from either the Nordic countries or mainland Europe, with only a few from other countries. The distribution across countries of origin were rather similar across generalists and experienced. Experienced birdwatchers and photographers dominated, with this category including a few guides/group leaders.

**Table 1:** Interviewed visitors by country/region across the three main groups of birding tourists

Type of visitor	Nordic countries^a^	Europe^b^	Great Britain^c^	USA and Australia	Sum
Generalist	11	9	3	2	25
Specialist birders	4	16	1	1	22
Specialist bird photographers	6	17		1	24
	21	37	4	4	71

^a^Finland, Norway, Sweden, Denmark

^b^Poland, the Czech Republic, France, Switzerland, Austria, Germany, the Netherlands, Spain, Belgium

^c^England, Wales, Scotland

### Observations

Most observed visitors did not behave in ways classified as illegal or inappropriate and potentially harmful. However, during the field work period, more than 40 events of illegal or potentially harmful behaviours were recorded. These observed incidents were grouped into six main categories: illegal and inappropriate, potentially harmful behaviour, as performed by generalist birdwatchers, experienced birdwatchers or experienced bird photographers (Table 2). Experienced bird photographers accounted for a proportionally higher share of the total number of incidents than generalists and specialist birders (Table 2).

**Table 2:** Overview of number of observed behaviours potentially disturbing birds

Type of tourist	Illegal behaviour	Inappropriate behaviour	Sum*
Generalists	6	7	13
Specialist birders	3	6	9
Specialist bird photographers	12	9	21
Sum	21	23	44*

*Behaviours based on the number of incidents, thus one visitor can be responsible for several incidents

The most frequently observed *illegal behaviour* was for visitors to continue past signs that delimit the areas designated for visitors. Crossing the ropes that mark the border for the designated area was observed less often. The most frequently observed *inappropriate behaviour* was general disturbance of birds nesting in the vicinity of the birdwatching shelter. The shelter was built for visitors; however, birds occasionally establish nests near or even within the shelter. Visitors sometimes also made abrupt movements or loud noises near these nesting birds, causing the birds to hesitate in walking past those visitors to their nests. Visitors seemed surprised to find nesting birds so close, and a couple were even pecked by the birds. Some visitors moved away, while others did not, and remained close to the birds.

Most observed incidents of potentially harmful behaviours were performed during the day with no obvious peak, e.g. during morning or evening. Neither were there any clear patterns suggesting that disturbing behaviours were more common when few or no other visitors were present (Table 3). Bystanders were seldom seen mimicking or copying depreciative behaviours of others.

**Table 3:** Number and percentage of observed behaviours potentially disturbing birds across time of day and social situation during the observation periods at Hornøya

Time of day/social setting	People present in immediate vicinity	People present in another part of the reserve	No people present in the same area	Sum (*n*)	Percent
Morning	3	6	1	10	23
Noon	7	9	4	20	46
Afternoon	4	5	2	11	26
Evening	1	1	0	2	1
Sum (*n*)	15	21	7	43	
Percent	35	49	16		100

### Perceptions of human-bird interactions and attitudes towards human disturbance of birds at Hornøya

Visitors saw seabirds as beautiful, interesting and having personalities. They used descriptors such as ‘unique’, ‘magnificent’, ‘incredible’ and ‘wonderful’, and some struggled to find the right words, feeling overwhelmed and surprised by the sheer number of birds. As in the work of Hill et al. (2014), the encounters with birds on Hornøya were characterised by contrasting and mixed feelings such as peacefulness and excitement. The experience appealed to multiple senses (views, smells, sounds) and had an emotional impact:

*Oh, it’s overwhelming! My senses are overwhelmed. Just, you know, kinda gleeful! I was in glee. So many birds. So many birds and then the beautiful landscape, the village right there, the water, the mountain.* (Woman, USA, generalist #42)

Reflections about landscape and notions of nature often accompanied comments about the birds. Informants were generally positively surprised that the birds were so close. In line with the findings in a study of whale tourism (Bertella 2016), few tourists worried about the close proximity to the animals.

The dominant belief expressed by informants was that one should respect nature and take care not to disturb the birds. Common opinions were that birds should come first, and that visitors must keep some distance and behave responsibly*.*

*Realistically I would like to hold the bird in my lap and kiss it and cuddle it. But I’m not going to. It’s not respectful and it’s terrible, it’s horrible. But I think most people are trying to follow the rules.* (Woman, USA, generalist #42)

The informants’ recognition of disturbance was often that it had occurred if the birds showed an obvious behavioural response and avoided or fled from visitors. A French birdwatcher said, ‘The definition of disturbing birds is if a bird leaves its nest because of us.’ However, when birds perceive danger they must evaluate the costs and benefits of leaving the nest due to the threat, or of enduring the stress and staying with their egg or chick (Reiertsen et al. 2018). Thus, some negative effects of disturbance such as increased stress are difficult to observe directly.

Nevertheless, some informants had a more nuanced way of thinking about disturbance. As one said:

*It’s not easy to answer this. Maybe you [divide] between slight disturbance and hard disturbance. The hard one is when you try to touch them and maybe the birds react really aggressive, and they maybe leave their nest. Not only temporary but stop breeding, or some of the nests get destroyed. It’s a hard disturbance. But if they only get a bit nervous and maybe fly away I don’t think it’s no problem, because they know how to come back.* (Man, Germany, specialist bird photographer #20)

Despite these reflections, this informant (#20) thought that ‘the birds absolutely don’t care about the visitors’ and did not believe visitors were having a negative impact on birds at Hornøya (#20). Others suggested that even if there were some disturbances, the resulting harm was limited. Many described the birds as being used to human visitors. Therefore, they believed there would be no serious negative long-term effects.

Overall, the attitudes expressed by informants towards disturbing birds were largely negative. However, although many of the informants at Hornøya evaluate disturbance of birds by visiting tourists as wrong and inappropriate, they believe its seriousness merits little concern: Disturbance is unacceptable, but on Hornøya, birds do not seem to care much about humans. A study of avian tourism in the Cantabrian Mountains of Central-Europe (Jiménez et al. 2011) found that birds over time were habituated to human activity in the vicinity. However, colony-breeding seabirds react differently than the species monitored in the Cantabrian Mountains (Jiménez et al. 2011) and their lack of shyness is not considered to be a result of habituation (Ellenberg 2017). The tendency to discount disturbance by visitors might also be a cognitive response resulting from a lack of knowledge (Manfredo 2008) about highly complex topics such as stress responses among colony-nesting seabirds.

### Normative beliefs

Norms function as ethical guidelines as to what is appropriate behaviour (Ajzen 1991). Informants generally expressed that one should respect the physical boundaries of the area designated for visitors, walk slowly and be quiet. Consequently, an encounter with birds will activate these norms and influence the behavioural response, for instance to step back if a bird suddenly comes close. Injunctive norms, in this case ‘what *should* be done’, were important to the informants. In contrast to injunctive norms, descriptive norms focus on ‘what others *do’*.

To understand more about the social setting at Hornøya, we examined informants’ perceptions about other visitors and their behaviour. The informants tended to categorise tourists into groups: ‘the typical tourist’, the ‘bird photographer’, or ‘the serious birdwatcher’ (see also Slater et al. 2019). Informants often created a prototypical member of each group, which shapes their beliefs about each group’s behaviour (Manfredo 2008). The typical unskilled birdwatching tourist is seen as a person with little prior knowledge about birds and limited experience of nature. In contrast, bird photographers are known to have advanced, expensive cameras and specialized clothing and gear, e.g. camouflage. A serious birdwatcher, equipped with an advanced telescope, high-end binoculars, and specialized birding clothing, e.g. a vest, perhaps taking field notes, might be interested in a specific group (taxon) of birds, or eager to see and add new species to his/her life list. These latter two groups agree with Curtin’s (2010) finding that self-representation by ‘serious wildlife tourists’ entails advanced skills, intellectual capital and specialized equipment.

At Hornøya, bird photographers were identified as the group most likely to break the norm of not crossing the boundary of the visitation area into the strict bird reserve, both in observations (Table 2) and interviews:

*[As] a photographer or bird photographer, we need to be closer. So, it’s a different way. We can spend much more time on one species to get enough time to be close enough, or to hide ourselves. So often we need to hide or get some installation. As a birdwatcher you can stay far away, they don’t really need a hide.* (Man, France, guide and specialised bird photographer #13)

There appears to be a conflict between eager birders and photographers: an us-versus-them mentality (Manfredo 2008). Photographers were accused of being more ignorant of the birds’ well-being:

*[Disturbance] doesn’t seem to be a problem … occasionally it seems to be more often photographers than birdwatchers who tend to get a bit closer just to get that perfect [photo]. Sometimes that can cause disturbance.* (Man, England, specialist; birdwatcher #18).

Generalist visitors had less professional equipment and talked about experiencing the bird island with all their senses, often observing birds with the naked eye, maybe taking simple distance photos with their mobile phone. These tourists were less tempted to perform inappropriate behaviour and were accused less by other interviewees of disturbing the birds. Some examples of behaviour that informants considered inappropriate but not illegal related to activities near the tourist shelter, where birds and humans occupied the same area, and some birds even took advantage of the tourist infrastructure by nesting under a bench. This situation confused some visitors.

Very few interviewees stated that they would tell someone to stop inappropriate or illegal behaviour. A Norwegian guide who had visited Hornøya many times thought that people visiting Hornøya were reluctant to express disapproval. One cause of this reluctance may be that people are wary of confronting rule-breakers, as those rule-breakers may have strong personalities and opinions. One informant said the following about situations in which an individual conducted illegal actions:

*A lot of people will say something about it, but not to the person. […] And people breaking rules are mostly people that are a bit more stronger and a bit more assertive, aggressive. We probably could, but we should react to it more often.* (Man, Belgium, generalist, #67)

An informant reasoned that ‘If I disrespect the line, I have already made the decision so I wouldn’t care about people’ (Man, Germany, specialist; birdwatcher #30). Nevertheless, this informant claimed he would approach a person behaving irresponsibly and ‘[…] ask him what he is doing or why. The main purpose here is not to take pictures, it’s to take care of the birds’ (#30).

Theory points out informal sanctions as important in creating a culture of appropriate behaviour in a conservation context (Guckian et al. 2018). This type of sanction can take a variety of forms, such as a scornful glance or a verbal admonition (Heywood 2002). The goal is to make the receiver uncomfortable so that they will adjust their behaviour quickly. Those most likely to correct others were professional guides or group leaders, who reacted to inappropriate behaviour by both their clients and by other visitors by, for instance, speaking to them or displaying strong body language.

Overall, this suggests there are social mechanisms in play to some extent at Hornøya, but they are not well developed either within or across the different segments of visitors. Heterogenous, most often self-organised visitor groups, speaking different languages, and sometimes slightly in opposition to each other combined with little organised guiding and a lack of inspector/ranger personnel present hinder development of shared social norms. Compounding these factors is the belief held by many visitors that, while disturbance should be avoided, the birds at Hornøya are not easily disturbed.

### Control beliefs and perceived behavioural control

Perceived behavioural control refers to *a visitor’s beliefs about the ability to undertake behaviour that provides the visitor with an experience of the birds that fulfils all expectations of the visit*. Put another way, behavioural control is how a single visitor assesses how easy or difficult it is to perform a certain behaviour. In the present context this might be a behaviour with the potential to cause disturbance or break formal or informal norms at Hornøya. The factors that influence perceived behavioural control are prior experience and information about the behaviour in question, observation of family and friends, and other factors that facilitate or hinder completion of the behaviour (Ajzen 2005; Miller 2017).

Self-reports from experienced birdwatchers and photographers often suggested that they had extensive knowledge about bird species and were able to assess the consequences of their own behaviour for the birds. A few informants admitted that they thought it was acceptable to challenge the guidelines and regulations to some extent, including the borders between legal and illegal visitation areas. Photographers often added that they were skilled at and concerned about finding the right light and angle for the pictures they sought. Many specialist photographers also stated that prior experience from birdwatching in other countries provided them with a solid basis for behaving correctly at Hornøya. Thus, both photographers and serious birdwatchers thought that they did not necessarily need to learn more. A French birdwatcher said, ‘Well, I’m a birder for so many tens of years that I don’t need that [education]’ (#52). A Swiss photographer highlighted his self-confidence by stating: ‘I can analyse the reaction of the bird. [….] It’s easy to recognise for a specialist. I must say, I’m a biologist! Hehehe! You understand a little bit more’ (Man, Switzerland, specialist; birdwatcher and photographer #54).

Strong perceived behavioural control, combined with a specific motivation to get close to birds, seems to be a major driver for behaviours that can disturb birds at Hornøya. Especially, in our study, specialist photographers seem prone to behave illegally. Strong self-confidence, previous experience from many bird-watching sites, and claimed knowledge of how to avoid disturbing birds, coupled with the attitude that the seabirds at Hornøya are not easily disturbed, paves the way for this ‘do as I want’ behaviour, that we judge as not straightforward ruthlessness (Ziegler et al. 2018), but behaviours in accordance with so-called responsibility-denial (Wirdner-Ward and Roggenbuck 2003).

### Attitudes towards management

Besides insight into the birdwatchers’ attitudes, norms, behaviour control beliefs and behaviour, the interviews provided many assessments and suggestions for how to improve management, both to stop illegal behaviour and to improve on the physical infrastructure to benefit both visiting birdwatchers and conservation values. First and foremost, the need for better signs and an improved fence delimiting the area prioritised for birds should be addressed. Better paths and information signs were repeatedly requested. As mentioned previously, visitors suggested that guides on the island could function as supervisors to correct illegal behaviour, while also improving the quality of the visit through nature interpretation. The significant potential of appropriate interpretation activities in wildlife tourism is highlighted by Moscardo et al. (2004).

Many participants eagerly reported about stricter measures implemented at other birding sites they had visited, through regulations of number of visitors and economic measures such as high fees. An American woman said: ‘I don’t mind the higher fee also to reduce the numbers – to price people out of coming here on purpose to keep the numbers down by raising the fee to eliminate people’ (#42). Raising fees can increase the exclusivity of the birdwatching product, which can in turn increase willingness to pay (Bertella 2016). Several also argued that stricter surveillance and inspections were necessary at Hornøya.

## Conclusion and implications

### Human behaviour potentially disturbing birds in a protected birdwatching site

This qualitative study of wildlife tourist behaviour at Hornøya adds to the literature on how to understand negative impacts on wildlife from humans conducting wildlife viewing. The typical motivation for visiting Hornøya was to have a unique experience with seabirds in their natural surroundings, without causing harm. Visiting birdwatchers were often emotionally affected by the bird encounters, which led to thoughts and reflections about environmental challenges and needs for nature protection. Disturbance of birds was generally unwanted and considered unacceptable. However, many visitors’ subjective interpretations of bird behaviour at Hornøya made them believe that the birds at the site are not easily disturbed. Such beliefs, in combination with weak social norms, lack of on-site inspectors and guide personnel, and strong perceived behavioural control among some visitor segments, led to occasional breaches of formal rules as well as incidents of inappropriate and potentially disturbing behaviours towards birds.

The behaviours identified at Hornøya that could disturb birds are likely partly wilful and partly unintentional (Wirdner-Ward and Roggenbuck 2003; Ziegler et al. 2018). However, both categories are presumably more common due to visitors’ lack of knowledge, which, again, might partly result from limited and insufficient management measures in the reserve, such as little detailed information and little presence of management personnel. Colony-breeding seabirds show few signs of disturbance (Ellenberg 2017; Reiertsen et al. 2018). Therefore, information and interpretation should specifically seek to explain how and why such wildlife can be disturbed even if they do not show it clearly (Curtin et al. 2009).

### Implications for biodiversity conservation and visitor management

The informants suggested many improvements and management measures to better combine bird conservation and valuable visitor experiences at Hornøya. Developing knowledge under the TPB framework could be useful to improve management since it systematically assesses attitudes, norms and perceived behavioural control (Gstaettner et al. 2017; Miller 2017). Further management measures could aim to build shared social norms among all visitors and reduce certain groups’ self-confidence in determining how to behave, by clear communication of a ‘code of conduct’ for all visitors to Hornøya. The findings further suggest that different management actions will likely be needed for different segments of the visiting birdwatchers. Education has the greatest effect on changing behaviour in those with the least knowledge, i.e. the ‘casual wildlife watchers’ (Cole and Scott 1999), whereas the ‘serious photographers’ conducting illegal behaviour might already be aware that they are breaching formal rules and therefore presence of surveillance personnel and stricter enforcement might be necessary for the latter group (Slater et al. 2019).

Better models for cooperation among conservation authorities, local authorities and tourism businesses should pave the way for funding presence of nature interpreters and rangers in the reserve during the main breeding and tourism season. Educational programmes can have a central role in preventing tourists’ depreciative behaviour, in addition to improving the quality of the visitor experience (Marion and Reid 2007). If necessary, more stringent approaches should be considered, including off-site tourism experiences such as live video streaming of nesting sites at a visitor centre at the harbour, and price/access regulations to keep the number of visitors at the island below a specified threshold.

### Further research needs

The findings in this study should be followed up with more quantitative approaches to quantify unwanted and illegal behaviour among different segments of birdwatchers through applying the TPB framework as well as other relevant conceptual understandings. Further research should also aim to understand how social norms could systematically be developed, e.g. through norm activation theory (Miller 2017) to be shared among varied segments of visitors. Additional research is also needed to consider why specialised and photography-oriented visitors might engage in illegal behaviour, despite their level of knowledge and stated pro-conservation attitudes. We also recommend studies that systematically assess how theory-based information- and interpretation measures can influence visitor behaviour on site in a more pro-environmental direction.

## Supplementary Information

Below is the link to the electronic supplementary material.

Supplementary file1 (DOCX 19 KB)

## Footnotes

1 The regulations have changed since this fieldwork was done, and audio recording of interviews is now considered as person-identifying data needing formal approval from NCRD (www.nsd.no/en).
