# Supplementary material for: Your place or mine? Exploring birdwatching tourists’ behaviour disturbing birds in a nature reserve
Source: Eur J Wildl Res. 2023 Apr 11;69(3):44. doi: 10.1007/s10344-023-01678-y (PMC10088662; doi:10.1007/s10344-023-01678-y)
Supplement: Supplementary file 1 — Supplementary file1 (DOCX 19 KB) [file 10344_2023_1678_MOESM1_ESM.docx]

Supplementary material: Interview-guide Hornøya

**Introduction**

I am a graduate student in nature-based tourism at the Norwegian University of Life

Sciences. We want to know more about the birding tourism at Hornøya, including the

visitor’s experiences. I would like to audio record the interview and ask for your consent to

do so. If you participate, you will remain anonymous, and your opinions stay confidential.

In my thesis, I might take out a few citations from the several interviews I will be doing this

summer. All citations will be anonymous. If you prefer to approve formally any anonymous

citation from this interview, I can send you the citation on email for approval. If so, I will need your email address.

**Background information**

1. Where are you from?

2. Have you been at Hornøya before?

3. What are your reasons for visiting Hornøya? Why do you find that interesting?

4. Where did you hear about this bird island?

5. What does it mean to you to be able to get good photos of the birds?

**Human-bird relation**

1. How did you experience your visit to Hornøya today?

2. What impressions did you get from the nature and wildlife here? How did it appeal to

your senses?

3. Were the sightings of the birds like you expected? What did you enjoy, and what did

you not like so much?

4. How do you think the birds reacts to the visitors at Hornøya?

5. Did you expect to have a close encounter with the birds? What do you define as a

close encounter?

6. If so, how did it make you feel?

7. Did you use a camera or binoculars today? What are the benefits of watching the

birds at Hornøya through a lens?

8. Do you prefer to watch with camera/binoculars or without? Why is that?

9. How would you describe the nature and landscape on Hornøya? Does it affect you in

any way?

10. What things came to your mind when walking around in the terrain? Any thoughts or

feelings?

**Behavioural beliefs**

1. Do you know where visitors are allowed to move around at Hornøya, and where they

are not supposed to be?

2. The designated area for visitors is marked by ropes and signs. What do you think are

the positive and negative sides being **outside** of the designated area?

3. What are the positive and negative sides by **being inside** the designated area?

4. What kind of human behaviour do you think can disturb the birds at Hornøya?

5. Have you experienced that the birds are disturbed by the visitors? If so, what makes you

think so?

6. Do you think birdwatching at Hornøya has negative consequences for the birds? If yes, how?

7. Do you think human presence at Hornøya can have positive outcomes for the birds? How?

**Normative beliefs**

1. Who do you think would not approve if you were to go outside of the designated area?

2. Would you worry about what others might think if you had gone outside the

designated area?

3. Did other people encourage or discourage you to go outside the area?

4. Do you think visitors respect the ropes and signs that show where it is allowed to be?

5. Did you see someone go past it? If yes, what do you think about that?

**Control beliefs**

1. On what basis do you think visitors should be allowed to walk outside the designated

area? Why or why not?

2. Did you learn about how to behave toward the birds before you arrived at Hornøya?

In general, or from local information (Tourist information, Vardø Havn, Wild

Varanger, Biotope)?

3. What do you think about the quality of the information in town and on Hornøya?

4. Do you think the area can be managed or regulated in a better way? Which measures

and for the benefit of the visitors or the birds?

5. What, if anything, do you think can be done to improve the visitor experiences?

**Additional opinions and viewpoints**

Is there anything else you would like to add to this interview and that we so far have not talked about?
